# Central Venous Line Placement prior to Gastric Bypass Improves Operating Room Efficiency

**DOI:** 10.5402/2012/816871

**Published:** 2012-07-08

**Authors:** D. Wayne Overby, Geoffrey P. Kohn, Karen J. Colton, Joseph M. Stavas, Robert G. Dixon, Anthony Passannante, Timothy M. Farrell

**Affiliations:** ^1^Department of Surgery, The University of North Carolina at Chapel Hill, Campus Box 7081, Chapel Hill, NC 27599, USA; ^2^Department of Radiology, The University of North Carolina at Chapel Hill, Campus Box 7510, Chapel Hill, NC 27599, USA; ^3^Department of Anesthesiology, The University of North Carolina at Chapel Hill, Campus Box 7010, Chapel Hill, NC 27599, USA

## Abstract

*Background*. Bariatric surgery has increased across America. Venous access is difficult in these patients. Anesthesiologists often utilize valuable operating room (OR) time acquiring reliable intravenous lines. Our objective was to determine if outpatient central venous line (CVL) placement improves OR efficiency and professional reimbursement for CVL insertion. *Methods*. In our bariatric practice, selected surgery patients have outpatient CVLs placed during prophylactic vena cava filter placement. In a cohort of 268 gastric bypass patients operated between 1/01 and 11/06, we compared time-to-incision between 106 with pre-established CVLs and 162 without. In addition, we determined professional compensation rates for CVLs placed outpatient versus CVLs inserted in the OR. *Results*. Patients with preoperative (outpatient) CVLs required 35.6 ± 12.5 minutes to skin incision compared with 42.5 ± 13.9 minutes for controls (*P* < 0.0001), and 34.9% had skin incision in <30 minutes compared with 16.4% of controls. Radiologists collected 28.2% of outpatient billings for CPT code 36556, compared with anesthesiologists who collected <1% when placing CVLs in the OR. *Conclusions*. Outpatient CVLs prior to gastric bypass improve efficiency in the OR with earlier skin incision. Professional reimbursement is better for outpatient CVLs than intraoperative inpatient CVLs.

## 1. Introduction

Obesity prevalence has increased over the last 20 years [1]. At the turn of the millennium, nearly two-thirds of Americans were overweight or obese, and almost 5% were morbidly obese [2]. Obesity shortens life expectancy and will likely soon be the leading cause of preventable death in the United States [3].

In the absence of reliable medical and behavioral therapies, and with the advent of minimally invasive technologies, bariatric surgery volumes expanded through 2003 [4–7]. More recent data suggest a leveling in procedure numbers despite the ongoing obesity epidemic [8], perhaps related to the evolution of center-of-excellence requirements [9, 10] and linked reimbursement systems [11]. Systems which restrict cases to high-volume centers may ensure better outcomes and reduced costs in certain environments but may also risk stifle small programs by proscribed requirements and limit national bariatric surgery capacity. The current reimbursement environment has set a narrow margin for surgeons and healthcare systems intending to provide state-of-the-art bariatric services [12].

Efficient delivery of operative care is necessary for the financial survival of bariatric programs. Therefore, programs have typically flourished in hospitals which focus on providing well-insured patients efficient customer-centric care [13]. Conversely, programs located in hospitals with prominent charitable and educational missions may struggle to preserve sufficient case volumes to maintain accreditation and profitability due to inherent inefficiencies [14].

Global efficiency initiatives for operating room processes have centered on improving case scheduling [15], data tracking [16], staff satisfaction [17], communications at all levels [18–20], and patient outflow [21]. At the provider level, many areas of inefficiency have been improved by simple tracking and delineation of accountability; however, certain patient-related variables, such as late arrival to hospital or difficult venous access, are more resistant to day-of-surgery systems improvement measures [22].

Bariatric surgery patients' body habitus may make achieving venous access challenging. When venous access efforts fail in the holding area, anesthesiologists often utilize valuable OR time acquiring reliable peripheral or central intravenous lines. These services may not be compensated and often impact an anesthesiologist's availability for other scheduled activity.

As a result of the increased prevalence of hypercoagulable states seen in the morbidly obese, we have initiated an aggressive program for venous thromboembolism prophylaxis in our bariatric patients, which includes liberal use of inferior vena cava (IVC) filters for a proportion of our patients who are deemed high risk for pulmonary embolism [23]. Filters are placed in the outpatient setting in the interventional radiology suite <24 hours prior to operation, with concomitant central venous line (CVL) insertion in all of these patients. Therefore, since some of our bariatric surgery patients arrive on the day of surgery with reliable venous access and others do not, we have a simple means to assess the effect of reliable venous access on operating room efficiency.

## 2. Methods

The study encompassed 268 consecutive patients who had laparoscopic gastric bypass between January 2001 and November 2006. Of these, 106 were referred to our interventional radiologists and had outpatient preoperative placement of a prophylactic IVC filter <24 hours prior to operation. These patients also received outpatient CVLs at the completion of the IVC filter placement.

All patients present through standard same-day-surgery channels on the day of operation. Routine preoperative protocols were applied by administrative, nursing, and physician staff. At University of North Carolina Hospitals, the precare nurses will attempt to achieve peripheral intravenous access if no access line is present. If venipuncture fails after two attempts, the anesthesiology team assumes responsibility for obtaining primary venous access. Patients are often taken from the holding area to the operating room under the direction of the attending anesthesiologist, and decisions about venous access and monitoring are made at his or her discretion.

We queried operating room databases to compare time between patient OR entry and skin incision (“in-to-skin”) for patients with and without outpatient CVLs. In addition, we searched billing databases for CVL collection rates. Since OR benchmarks for “in-to-skin” times are typically stratified by 15-minute increments, we graphically depicted the subject and control data in this fashion. Statistical comparison utilized the raw continuous data was made by the nonpaired *t*-test, using *P* < 0.05 as a measure of statistical significance. Times were described as arithmetic means ± standard deviations.

## 3. Results

The 106 patients who received preoperative CVLs had demographics and average BMIs similar to the 162 patients who did not receive preoperative CVLs (Table 1).

**Figure 1:**
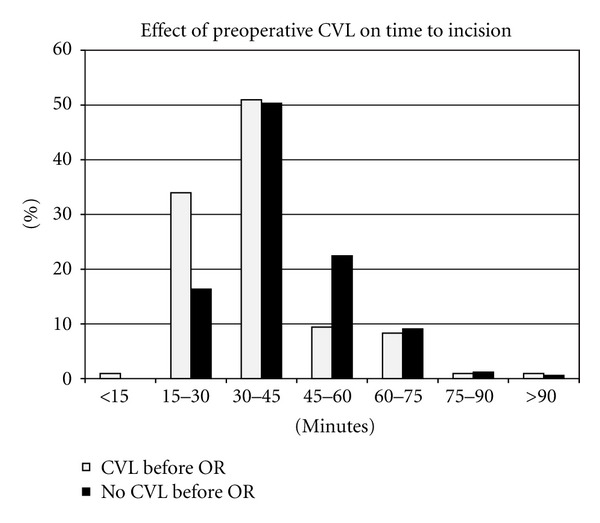


Patients with preoperative CVLs had mean “in-to-skin” time of 35.6 ± 12.5 minutes versus 42.5 ± 13.9 minutes for those without preoperative CVLs (*P* < 0.0001). When assessed in quarter-hour increments, the presence of a preoperative CVL was associated with 34.9% of subjects having skin incision by 30 minutes versus 16.36% of controls (Figure 1).

Regarding reimbursement, interventional radiologists performing the outpatient CVLs collected 28.2% of billings for CPT code 36556. In contrast, those anesthesiologists who placed intraoperative CVLs in the operating room collected less than 1% of dollars billed.

Among those 106 patients who received preoperative CVLs, one catheter-related infection occurred in a patient with a long hospitalization from respiratory failure, and two self-limited catheter site infections were noted after CVL removal.

## 4. Discussion

While the number of American adults suffering from obesity continues to increase, the annual number of bariatric operations has been stable or decreasing in recent years [8]. This trend may be attributable to the evolution of programs designed to direct patients to certified high-volume centers where the quality of care is believed to be best. With the arrival of a new administration in our nation's capital, America has again focused awareness on prevention, management of chronic disease, and cost containment [24]. Bariatric surgery will likely come to increased attention as the only contemporary option to decrease the burden and overall utilization of healthcare resources related to America's most costly disease. If governmental interventions crystallize candidacy criteria and eliminate bariatric surgery exclusions among healthcare insurers, the resultant resurgence in demand for procedures may tax the capacity of the system to increase patient throughput.

Increasing efficiency of systems is a strategy for increasing delivery of care without a concomitant and proportional increase in cost. In the highly regulated environment of bariatric surgery, efficiency initiatives will no doubt be integral in early efforts to maximally utilize existing systems, at least until the projected rising demand drives further program and infrastructure development.

This study highlights one obvious efficiency intervention related to perioperative intravenous access. We have demonstrated that outpatient CVL placement in planned bariatric patients improves efficiency on the day of surgery. When the effect of a small incremental OR time savings is considered on a broader scale, the potential to achieve significant monetary savings while perhaps even enhancing effective capacity emerges. With case volumes driving all systems for bariatric program certification, such efficiencies may bring many smaller centers to case levels that allow credentials to be obtained, with improved ability to compete for patients and contracts. In addition, improved reimbursement rates for CVL implantation noted in our study will further support the financial success of a program.

Although a higher level of difficulty for intravenous access in morbidly obese patients has not been established in comparative studies, there are a number of references to obesity as a contributing factor in difficult peripheral and central intravenous access [25–31]. Our data suggest that difficult venous access in bariatric surgery patients may contribute to OR delays.

With operating room cost per minute on the order of $15–25 [32] and with an average $66 per minute charged to patients, [33] a savings of 15 minutes per case can have a significant financial impact at the hospital level, especially when a single attending anesthesiologist oversees two, three, or even four rooms at one time. Moreover, each such delay avoided will prevent accumulating costs for subsequent patients' scheduled in the same operating room. Finally, the above estimates of cost do not account for lost efficiencies in terms of underutilized surgeons, operating room and recovery room personnel, and inpatient resources. Since anesthesiologists are reimbursed less than 1% for CVLs placed in the OR, it is clear that these providers have no financial reason for resisting program initiatives to move CVL placement to the outpatient setting.

In addition to improved OR efficiency, we believe there are likely other intangible benefits to CVL placement prior to gastric bypass. A preexisting CVL reduces the number of tasks that must be performed prior to the induction of general anesthesia, which may decrease frustration levels when dealing with potentially difficult morbidly obese patients. The inpatient nursing and phlebotomy staff also benefit from secure venous access for delivery of medications and blood draws, which decreases their frustration and improves efficiency and customer service during the inpatient stay. Finally, patients are happy to avoid multiple unsuccessful phlebotomy and peripheral venous line (PVL) placement attempts.

There are risks associated with CVLs, including those associated with placement such as arterial puncture, hematoma, and pneumothorax, and those which occur after placement such as catheter-related infections, catheter malfunction, and catheter-related thrombosis. Since the CVL catheters described in this study were placed using ultrasound guidance by experienced vascular interventional radiologists, the insertion complication rate was low (0.62%) [34], and since the majority of patients were immune competent, not critically ill, and had brief CVL dwell times, low rates of catheter-related infection were seen. In fact, similar risks have been associated with PVLs, including arterial puncture, hematoma, infiltration, catheter malfunction, and catheter-related infections. While the rate of minor complications such as infiltration or phlebitis is high with PVLs (up to 21.7%) [35], the rate of serious complications such as bacteremia is probably lower when compared to CVLs.

Anecdotal reports of increased patient and anesthesiologist satisfaction with CVLs are being assessed in subsequent studies. Additional implementation of a plan for increased CVL utilization to enhance OR efficiency will need to be balanced against the impact on interventional radiology unit capacity and professional satisfaction of the affected radiologists, as well as the impact on trainees in the operating rooms.

One drawback of the current study is its retrospective nature. Useful data regarding OR efficiency, related costs, potential savings, and complications could be obtained from a prospective randomized control trial comparing CVL placement prior to gastric bypass to CVL placement at the time of gastric bypass to peripheral intravenous access at the time of gastric bypass.

## 5. Conclusions

Outpatient placement of CVLs prior to gastric bypass improves the efficiency of the operating room with earlier skin incision. For patients having IVC filter placement prior to bariatric surgery, concomitant CVL placement is advised.

For those patients not referred for IVC placement, we suggest surgeons and anesthesiologists evaluate for difficult intravenous access during the outpatient preoperative visit and consider referral for ultrasound-guided access during the 24 hours prior to scheduled surgery. Such efforts will improve day-of-surgery efficiency and professional reimbursement for necessary procedures.

## Figures and Tables

**Table 1:** 

	*n*	Percent female	BMI ± SD (kg/m^2^)
Patients with CVL	106	84	51.6 ± 9.0
Patients without CVL	162	86	50.9 ± 8.8
*P* value			NS
